# Navigating the Controversies: Role of TRPM Channels in Pain States

**DOI:** 10.3390/ijms251910284

**Published:** 2024-09-24

**Authors:** Maria A. Gandini, Gerald W. Zamponi

**Affiliations:** 1 Department of Clinical Neurosciences, Hotchkiss Brain Institute, Cumming School of Medicine, University of Calgary, Calgary, AB T2N 1N4, Canada; zamponi@ucalgary.ca; 2 Department of Clinical Neurosciences, Alberta Children’s Hospital Research Institute, Cumming School of Medicine, University of Calgary, Calgary, AB T2N 1N4, Canada

**Keywords:** TRPM2, TRPM3, TRPM8, analgesia, nociception, G-protein coupled receptors

## Abstract

Chronic pain is a debilitating condition that affects up to 1.5 billion people worldwide and bears a tremendous socioeconomic burden. The success of pain medicine relies on our understanding of the type of pain experienced by patients and the mechanisms that give rise to it. Ion channels are among the key targets for pharmacological intervention in chronic pain conditions. Therefore, it is important to understand how changes in channel properties, trafficking, and molecular interactions contribute to pain sensation. In this review, we discuss studies that have demonstrated the involvement of transient receptor potential M2, M3, and M8 channels in pain generation and transduction, as well as the controversies surrounding these findings.

## 1. Introduction

The physiological process in which pain becomes a conscious experience is termed nociception. Nociception, from the Latin word “nocere”, meaning to harm or hurt, serves an evolutionarily adaptive function where potentially dangerous stimuli are detected [1]. Nociception involves transducing sensory information into electrical signals by distinct groups of primary sensory neurons, each specializing in specific modalities. This cascade of events triggers the release of neurotransmitters and neuropeptides, which activate ascending projections to the brain where pain is perceived as an unpleasant sensation [1,2,3]. Under pathological conditions such as inflammation or tissue injury, these nociceptors trigger hypersensitivity to mechanical and thermal stimuli, resulting in phenomena like allodynia (pain triggered by non-painful stimuli), hyperalgesia (heightened sensitivity to pain, including an exaggerated response to it), or spontaneous pain in the absence of any apparent stimulus [1,2,3].

Nociceptors are characterized by a pseudo-unipolar axonal projection. They have their cell bodies in the dorsal root ganglia (DRG) or trigeminal ganglia (TG, for nerves that innervate the head), and their axons bifurcate into two branches. The first branch extends as a lengthy projection with peripheral endings within the skin and internal organs. In contrast, the second branch establishes a connection to the central nervous system (CNS) in the spinal cord or brain stem (for the TG neurons) [4].

Axons of primary afferent neurons are divided into three main groups based on their conduction velocity and the types of stimuli that they primarily detect, although all subtypes serve multiple sensory functions. Aβ fibers are fast-conducting (>30 m/s) and myelinated, with a low threshold for activation. They primarily respond to non-painful tactile stimuli, such as light touch and mild thermal changes, and are involved in reflexive responses. Aδ and C fibers are primarily nociceptors, with higher activation thresholds, meaning that they mainly respond to potentially harmful stimuli that could cause tissue damage. Aδ fibers are thinly myelinated and conduct at moderate speeds (2–30 m/s), while C fibers are unmyelinated and conduct more slowly (≤2 m/s) [1,2,3,4].

Traditionally, DRG neurons have been classified based on their size, degree of myelination, and nerve conduction velocity, among other properties. However, the function of different peripheral sensory neuron subtypes is ultimately determined by the expression of specific genes. Although significant efforts have been made to characterize the molecular features that define each subtype, identifying specific markers for distinct subpopulations of nociceptive neurons has been challenging. However, Buhuyan and colleagues [5] recently created a harmonized DRG neuronal reference atlas containing 18 neuronal types, of which 15 subtypes have at least one known function attributed to them from rodent studies. These studies utilized knock-in mice expressing Cre recombinase for direct genetic access to the cell types (see Table 1).

This classification creates a molecular framework that defines peripheral sensory neuron cell types, enabling the mapping of specific functions to each type.

On the other hand, it is well known that neuroinflammation resulting from the interplay between immune cells and neurons plays a pivotal role in pathological pain. Resident and circulating immune cells migrate and become activated in response to peripheral tissue damage, inflammation, or nerve injury [6]. Inflammatory mediators, including proinflammatory cytokines, chemokines, and reactive oxygen/nitrogen species released by these cells, can induce nociceptor sensitization and enhance the excitability of nociceptive primary afferent neurons, a phenomenon termed peripheral sensitization [7]. The persistent hyperexcitability of peripheral nociceptive neurons initiates synaptic facilitation and enhances the responsiveness of dorsal horn neurons in a process known as central sensitization [7]. Interestingly, the activation of spinal glial cells, particularly microglia, contributes to this process by producing pronociceptive inflammatory mediators [8].

## 2. Transient Receptor Potential (TRP) Channels

A fascinating feature of nociceptors is that they can respond to a combination of stimuli. This versatility is linked to the expression of specific receptors from the transient receptor potential (TRP) ion channel family. Moreover, due to their polymodal nature, TRP channels, since they can be activated by physical factors (such as temperature, voltage, and pressure) and chemical stimuli [9], also play a key role in different immune and glial cells [10]. The TRP channel family is divided into seven subfamilies based on sequence similarity. Most of them are non-selective cation channels [11]. There are 27 cationic TRP channels present in humans, sub-divided into six families: canonical (TRPC), vanilloid (TRPV), melastatin (TRPM), ankyrin (TRPA), mucolipin (TRPML), and polycystic (TRPP) [11,12,13].

All TRP channels share a common topology and, as with other voltage-gated ion channels, are composed of six transmembrane segments per subunit with intracellular N- and C-termini. The putative S5 and S6 segments of these subunits, upon tetramerization, form a central ion-conducting pore, featuring a non-selective cation selectivity filter [11,12]. Although sharing a similar structure, TRP channels are remarkably diverse since they feature different molecular domains that regulate ion permeation, tetramerization, ligand binding sites, and modulation by intracellular signaling molecules, giving them specific activation mechanisms and cellular functions.

The TRP channel family has three crucial functional properties that can be altered to regulate their gating—activation, sensitization, and desensitization. Activation involves a stimulus allowing channel transition from a closed to open state, allowing ion conduction across the membrane. This results in changes in membrane potential and, if the stimulus is strong enough, the generation of action potentials. Sensitization is when the channel’s activity is potentiated in response to various stimuli or regulatory molecules, making it respond more efficiently. Desensitization happens when the channel becomes unresponsive to stimuli by adopting a conformation where there is no ion permeation [14].

In this review, our attention will be directed toward exploring the involvement of the TRPM channel subfamily in inflammation and neuropathic pain. As such, our focus will be on TRPM2, TRPM3, and TRPM8 channels.

## 3. The TRPM Channels Subfamily

The TRPM subfamily consists of eight members, from TRPM1 to TRPM8. The original member TRPM1 was discovered through the identification of transcripts enriched in human melanomas, hence its initial name, Melastatin. Based on amino acid sequence similarity, the human TRPM gene family can be categorized phylogenetically into two groups: TRPM1, TRPM3, TRPM6, and TRPM7, and TRPM2, TRPM4, TRPM5, and TRPM8 [15,16].

**Figure 1:**
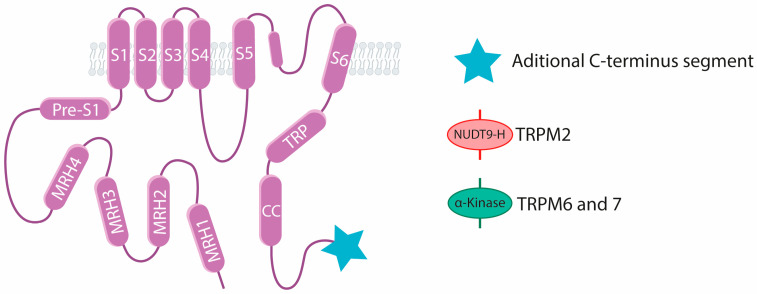
Topology of TRPM channels. TRPM channels have 4 melastatin-homology regions (MRH) followed by a pre-S1. The S1–S6, α-helices form the transmembrane-spanning channel segments. Pore is located between S5 and S6. The C-terminus contains the TRP and the coiled-coil (CC) domain. Additionally, TRPM2 channels have a NUDT9 homology (NUDT9-H) domain, and TRPM6 and TRPM7 have a serine/threonine protein kinase domain (α-Kinase) in their C-terminus. Figure modified from [16].

All TRPM channels are comprised of four domains termed Melastatin Homology Regions (MHR1–MHR4) and one homology region or pre-S1 domain in their N-terminus (Figure 1). The C-terminus features the TRP box, positioned almost parallel to the membrane surface, which exhibits a highly conserved amino acid sequence crucial for channel stability within the plasma membrane. The TRP box is followed by a coiled-coil domain, which is important for tetrameric complex assembly of channel subunits and pore gating. TRPM2, TRPM6, and TRPM7 contain additional C-terminus segments with enzymatic activity. TRPM2 has a nucleoside diphosphate pyrophosphatase (a NUDT9 homology) domain, while TRPM6 and TRPM7 have a serine/threonine protein kinase (α-kinase) domain [15,16]. Out of the 8 members of the TRPM subfamily, TRPM2 [17,18,19,20,21,22], TRPM3 [23], TRPM4 [24,25,26,27], TRPM5 [28], TRPM7 [29,30], and TRPM8 [31,32,33] structures have been elucidated through cryo-electron microscopy (cryo-EM).

TRPM channels undergo extensive alternative mRNA splicing, resulting in a diverse array of structural variants with varying biophysical properties and, in some cases, leading to non-functional channel isoforms. For instance, the TRPM3 channel has the largest number of different splice variants among all the TRP family members, exhibiting different ionic selectivity, some favoring monovalent cations while others prefer divalent ions [34,35,36].

Activation mechanisms within the TRPM subfamily vary significantly. Intracellular Ca^2+^ and Mg^+^, membrane potential, and membrane phospholipid phosphatidylinositol 4,5-bisphosphate (PIP2) are key regulators [37,38]. Moreover, half of its members (TRPM2, TRPM3, TRPM4, TRPM5, and TRPM8) exhibit sensitivity to a broad spectrum of temperatures, ranging from cold to hot, leading to them being referred to as thermoTRPs [39,40,41,42].

### 3.1. TRPM2

TRPM2 exhibits widespread expression throughout the body, including in the peripheral and central nervous systems, heart, pancreas, liver, spleen, and immune cells [16]. Functionally, TRPM2 permeates both Na^+^ and Ca^2+^. Its activation requires adenosine diphosphate ribose (ADPR), intracellular Ca^2+^, and PIP2. The NUDT9 domain of TRPM2, in the presence of Ca^2+^, binds ADPR to promote channel opening [43]. Due to this mechanism, TRPM2 is categorized as a “chanzyme”, indicating a channel with enzymatic activity, a characteristic shared with two other members, TRPM6 and TRPM7. TRPM2 is considered a chanzyme due to its ability to cleave ADPR, converting it into adenine monophosphate (AMP) and ribose-5-phosphate [44]. Additionally, TRPM2 functions as a thermoTRP, capable of sensing physiological body temperatures (37–40 °C). Interestingly, metabolic factors such as redox signals have been reported to sensitize the channel and regulate its temperature-sensing properties [45].

Most of the knowledge about the role of TRPM2 in pain states comes from TRPM2 knockout (KO) mice. In the first series of experiments, Haraguchi et al. [46] did not find differences in withdrawal thresholds to mechanical stimulation or thermal nociceptive latency between the WT and TRPM2 KO mice, which means no difference in basal sensitivity. However, when an acute inflammatory pain model (carrageenan-induced paw edema) or a neuropathic pain model (partial-sciatic nerve ligation, pSNL) were used, mechanical allodynia and thermal hyperalgesia were significantly reduced in TRPM2 KO mice. Moreover, they found that TRPM2 KO has an impaired inflammatory response due to a decreased CXCL2 production from both resident and recruited macrophages, thereby reducing neutrophil infiltration at the inflamed sites. Although CXCL2 itself does not induce pain [47], infiltrating neutrophils play a pivotal role in the generation of inflammatory pain [48]. Interestingly, TRPM2 KO mice presented reduced responses in the acetic acid-induced writhing behavior test (recognized as an acute inflammatory pain model) compared to WT mice. Mechanical allodynia was also decreased in TRPM2 KO mice across various pain models, including monosodium iodoacetate-induced osteoarthritis, experimental autoimmune encephalomyelitis, paclitaxel-induced peripheral neuropathy, and streptozotocin-induced painful diabetic neuropathy when compared to WT mice.

TRPM2 is functionally expressed in DRG neurons [49]; however, TRPM2 KO did not present any difference compared to WT animals on TNF-α, H_2_O_2_, or capsaicin-evoked hypersensitivity. Since some inflammatory cytokines, such as TNF-α, and reactive oxygen species produced from activated macrophages and other immune cells elicit pain by directly acting on nociceptive neurons [50,51,52], it was suggested that TRPM2 is not involved in nociceptor sensitization. Altogether, these results show that TRPM2 plays a role in various pathological inflammatory and neuropathic pain pathways, yet its involvement in nociceptive pain appears to be limited.

It is worth mentioning that TRPM2 KO mice show suppression of spinal microglia activation following nerve injury in the spinal cord. Furthermore, cultured macrophages and microglia derived from TRPM2 KO mice showed a significant decrease in CXCL2 production and inducible nitric oxide synthase induction. These findings collectively suggest that TRPM2 expression in macrophages and microglia exacerbates peripheral and spinal inflammatory responses.

**Figure 2:**
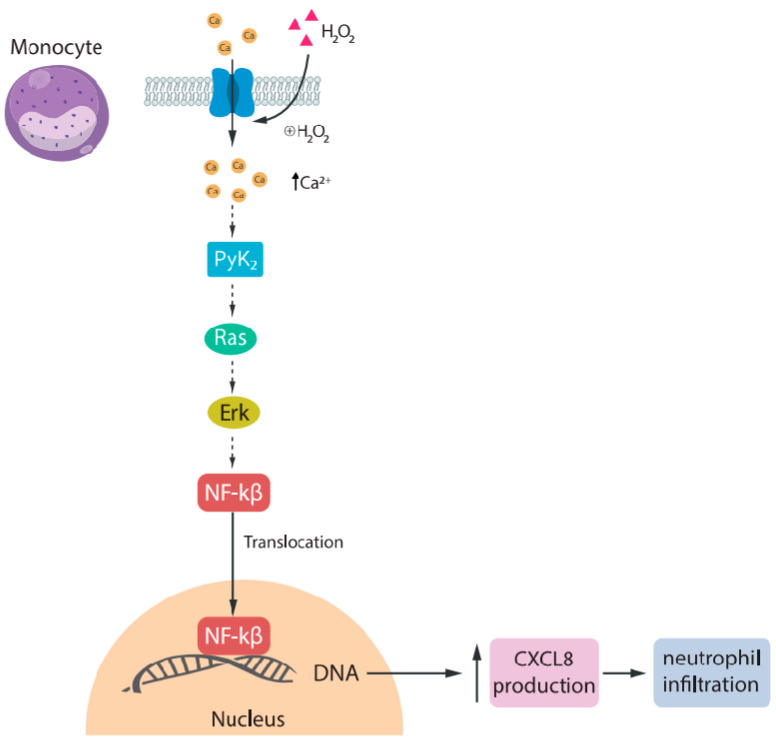
Activation of TRPM2 induces CXCL8 production. In monocytes, TRPM2 channels, when activated by H_2_O_2_, allow the influx of Ca^2+^, which activates PyK2, Ras, ERK, and finally the transcription factor NF-κB. NF-κB is translocated to the nucleus and increases the transcription of CXCL8. This promotes neutrophil infiltration to the injury site.

The role of TRPM2 in immune cells has been investigated. Yamamoto et al. [53], using human U937 monocytes, demonstrated that TRPM2 mediates Ca^2+^ influx triggered by H_2_O_2_, amplifying downstream Ras and Erk signaling via the Ca^2+^-dependent tyrosine kinase Pyk2. This cascade leads to the nuclear translocation of NF-κB and the production of CXCL8 (Figure 2). Notably, monocytes from TRPM2 KO mice displayed diminished CXCL2 production, exacerbating dextran sulfate sodium (DSS)-induced ulcerative colitis. CXCL2, along with CXCL8 in humans, serves as a primary inducible chemokine responsible for orchestrating neutrophil infiltration and consequent tissue damage across various animal models of inflammation and injury [54]. Interestingly, TRPM2 KO neutrophils retained their migratory capabilities. Moreover, Wehrhahn and colleagues [55] observed an upregulation of TRPM2 channels when incubating human primary monocytes with lipopolysaccharide (LPS), a cell wall component of Gram-negative bacteria known to contribute to bacterial toxicity. Acute down-regulation of the expression of TRPM2 channels via short hairpin RNAs resulted in a decrease in LPS-induced production of IL-6, IL-8, IL-10, and TNFα. Taken together, these results suggest that the influx of Ca^2+^ mediated by TRPM2 in monocytes triggers the production of proinflammatory cytokines and chemokines, promoting the infiltration of neutrophils and thereby intensifying inflammation.

In highly contrasting results, other studies have linked the lack of TRPM2 to worsening inflammatory responses. It has been reported that TRPM2 KO mice exhibit increased production of proinflammatory cytokines CXCL2, TNFα, and IL-6 and have poorer survival rates in response to LPS compared to WT animals [56] through a mechanism that includes NADPH oxidase and ROS production inhibition, resulting in a negative feedback mechanism where TRPM2 activation buffers LPS-induced ROS and proinflammatory activity [57]. Moreover, another study showed that inhibiting TRPM2 was associated with LPS-induced inflammation, neutrophil migration, and neutrophil-mediated vascular injury through a mechanism independent of TRPM2 cation channel activity [58]. The authors showed that LPS-induced ROS in neutrophils negatively regulates neutrophil migration by oxidizing TRPM2 at Cys549 of its N-terminal region, triggering the interaction and internalization of the chemoattractant receptor FPR1, a key regulator of the inflammatory environment [59].

The role of TRPM2 in the immune system seems to be complex, exhibiting both proinflammatory and anti-inflammatory functions. Further research is needed to address the discrepancies observed among different models and establish a clear role of TRPM2 in the immune system and how such cellular functions relate to pain signaling.

### 3.2. TRPM3

**Figure 3:**
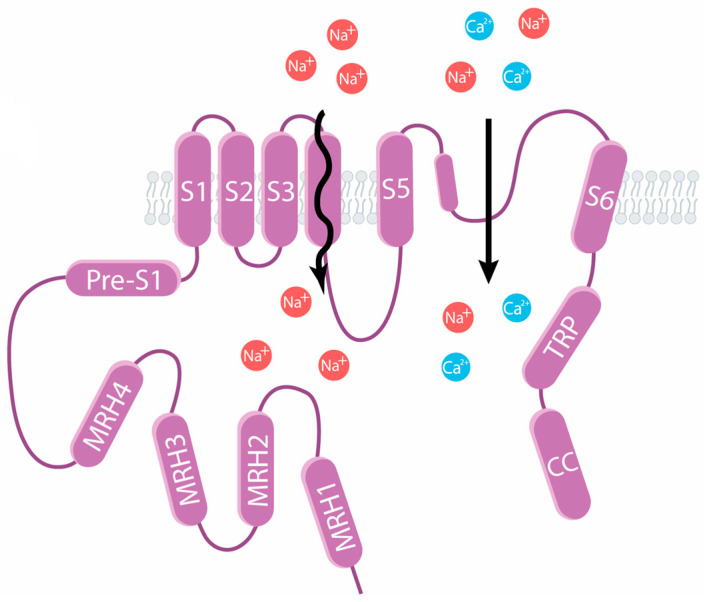
TRPM3 channels have two permeation pathways. The canonical pathway is located at the pore of the channels and transports Na^+^ and Ca^2+^ ions, and the alternative pore is located at the S4 segment where Na^+^ ions are transported.

TRPM3 channels are highly expressed in nociceptors of mice, rats, and humans, where they play a key role in the response to heat. They can also be found in oligodendrocytes, pancreatic β-cells, vascular smooth muscle cells, adipocytes, retina, and distinct parts of the brain, among other tissues [16]. TRPM3 channels can be activated by ligands, heat, and membrane depolarization [60,61,62]. TRPM3 possesses two different permeation pathways: (1) the canonical pathway activated by heat or pregnenolone sulfate (PS, an endogenous excitatory neurosteroid) and (2) an alternative pore (“omega-like” current), activated by co-stimulation of PS and the antifungal drug clotrimazole (Clt) or the synthetic compound CIM0216 (which opens both permeation pathways) [63,64] (Figure 3).

As mentioned in the introduction, TRPM3 exhibits extensive alternative splicing. Most of these isoforms are classified into TRPM3α and TRPM3β groups. TRPM3α isoforms start with exon 1 and exclude exon 2, while TRPM3β isoforms start with exon 2 [60,65,66]. One of these splice variation sites includes a 12 amino acid difference in the pore-forming loop between isoforms TRPM3α1 and TRPM3α2 [60,67], which leads to significant changes in their biophysical properties. TRPM3α2 are Ca^2+^ channels with permeability to other divalent cations like Zn^2+^ and Mg^2+^. In contrast, TRPM3α1 presents reduced permeability for divalent cations and high selectivity for monovalent cations [60,68]. Moreover, the length of the pore loop in TRPM3 splice variants plays a crucial role in defining the pharmacological properties of the channels. For instance, the long pore loop variant TRPM3α1 is insensitive to PS, and its heat sensitivity is lost [35]. Although many isoforms have been reported, their physiological role is still not well documented; however, it is worth mentioning that TRPM3 channels in different tissues exhibit similar pharmacological and biophysical properties with TRPM3α2 [35,60,69]. Thus, in the following sections, the term “TRPM3 channels” reflects predominantly the TRPM3α2 variant.

To investigate the role of TRPM3 in nociception, Vriens et al. (2011) injected PS into the hind paws of WT and TRPM3 KO mice. The injection provoked nocifensive responses, measured as paw licking and lifting, in WT but not in KO animals. Additionally, when the animals were injected with CFA (complete Freund’s adjuvant) (Vriens 2011) or carrageenan [70] in the hind paw, TRPM3 KO mice did not develop thermal hyperalgesia. Krügel et al. [71] found that treating animals with the anticonvulsant primidone, a TRPM3 inhibitor, either as a co-injection with PS or administered systemically before injecting PS in the hind paw, significantly attenuated nocifensive responses to chemical pain induced by TRPM3 activation. Moreover, intrathecal injection of the TRPM3 agonist CIM0216 reduced paw withdrawal latency to radiant heat in WT but not in TRPM3 KO mice [70]. Systemic pretreatment with primidone or ononetin (another potent TRPM3 inhibitor) prevented heat hyperalgesia in the CFA animal model [71,72], and a similar response was observed in TRPM3 KO mice [61].

Intraperitoneal administration of TRPM3 blockers [73], the flavonoids liquiritigenin [74], and isosakuranetin [75] alleviated mechanical, thermal, and cold hyperalgesia in a neuropathic pain model (chronic constriction injury (CCI) of the sciatic nerve) in rats. Furthermore, in rats pretreated intrathecally with TRPM3 inhibitors (isosakuranetin, naringenin, and ononetin) before receiving an intrathecal injection of N,N-dimethylsphingosine (DMS), there was an attenuated development of mechanical hyperalgesia. TRPM3 RNA has been detected in the spinal cord through RT-PCR [76] and by single-cell RNA in dorsal horn neurons [77,78] and spinal cord astrocytes [78]. However, these experiments demonstrate that TRPM3 is functionally expressed on the central terminals of nociceptive nerve fibers and that its inhibition suppresses spinal mechanisms contributing to DMS-induced mechanical pain hypersensitivity [79].

In contrasting results, Su et al. [70] tested two TRPM3 inhibitors, primidone and isosakuranetin. They found that while both inhibitors were consistently effective in reducing heat hyperalgesia, they did not affect mechanical or cold hyperalgesia in CCI mice, whether administered in the hind paw or intrathecally. Interestingly, intraperitoneal injection of isosakuranetin at 2 mg/kg, but not at 0.5 mg/kg, inhibited cold and mechanical hypersensitivity in CCI in both WT and TRPM3 KO mice, thereby revealing a dose-dependent off-target effect. Intraperitoneal isosakuranetin also inhibited spontaneous pain-related behavior in CCI in the conditioned place preference assay, and this effect was not observed in TRPM3 KO animals. Interestingly, inhibitors led to paw withdrawal latencies that remained lower than those of TRPM3 KO mice, regardless of dose or route of application. However, in sham-operated animals, both drugs increased paw withdrawal latencies to levels comparable to those seen in TRPM3 KO mice. This raises the question of whether different splice isoforms are expressed during nerve injury, constituting a regulatory mechanism for TRPM3 expression that may not be reflected in KO animals. Additionally, the Trpm3 gene encodes the microRNA miR-204 in intron 9 in both humans and mice, so miR-204 should be considered when investigating TRPM3 functions [66,80].

Lastly, in a model of chemotherapy-induced peripheral neuropathic pain (CIPNP), mice injected with a single dose of oxaliplatin developed cold and mechanical hypersensitivity, an effect not seen in TRPM3 knockout (KO) mice. Systemic injection of isosakuranetin (2 mg/kg, a dose determined to be off target by Su et al. [70]) resulted in a significant increase in mechanical threshold and a decrease in the cold nociceptive response. In a different study, increased expression of the TRPM3 transcript after oxaliplatin treatment was observed; however, the authors did not find an increase in TRPM3 activity using calcium imaging experiments [81]. Taken together, it seems that the role of TRPM3 in oxaliplatin-induced peripheral neuropathic pain is still not completely well established.

There are few reports examining the regulation of expression and functional activity of TRPM3 channels. Su et al. suggested that TRPM3 channels have spontaneous activity, as TRPM3 KO or the use of TRPM3 channel inhibitors reduced the increase of c-Fos and pERK (neuronal activity markers) in DRGs and the spinal cord of CCI animals. Remarkably, when the alternative permeation pathway (“omega-like current”) in addition to the central calcium-permeable pore is activated, there is an increase in spike frequency in DRG neurons and an increase in paw licks and lifts when injected with PS and Ctl [63].

In 2020, Mulier and colleagues [82] compared mRNA expression and functional activity of TRPM3, TRPV1, and TRPA1 in the CFA mouse model. They found that CFA injection into hind paws triggered increased TRPM3 expression in DRG neurons without changes in TRPA1 or TRPV1 levels. Inflammation increased the activity of all three TRP channels, evidenced by increased Ca^2+^ responses to specific agonists in neuronal cell bodies and nerve endings. Interestingly, pharmacological inhibition of TRPM3 eliminated Ca^2+^ responses to TRPM3 agonists and reduced responses to TRPV1 and TRPA1 agonists in neurons innervating the inflamed paw. These results suggest that tissue inflammation leads to a functional upregulation of all three molecular heat sensors that are all involved in the pain response to noxious heat.

Following a chronic constriction injury to the infraorbital nerve in rats, a model of trigeminal neuralgia, they observed an increase in the expression of TRPM3 and TRPV4, another member of the TRP family, as evidenced by using Western blotting and immunochemistry. Interestingly, the injection of botulinum toxin type A, a neurotoxin that cleaves synaptosomal-associated protein of 25 kDa (SNAP-25), reversed the increased expression, and the pain threshold measured by Von Frey hairs [83]. Since no pharmacological studies were conducted, the specific contribution of each channel to mechanical hyperalgesia remains unclear. However, it has been reported that the cleavage of peripheral SNAP-25 modulates TRPV1 mobilization from intracellular stores through regulated exocytosis [84,85] and reduces total TRPV1 expression by inhibiting its trafficking to the plasma membrane and making TRPV1 susceptible to ubiquitination and subsequent proteasomal degradation [86]. This finding opens the door to an unexplored area of research, specifically the trafficking of TRPM3 and how they interact with the exocytotic machinery to regulate their expression when sorted into vesicles.

**Figure 4:**
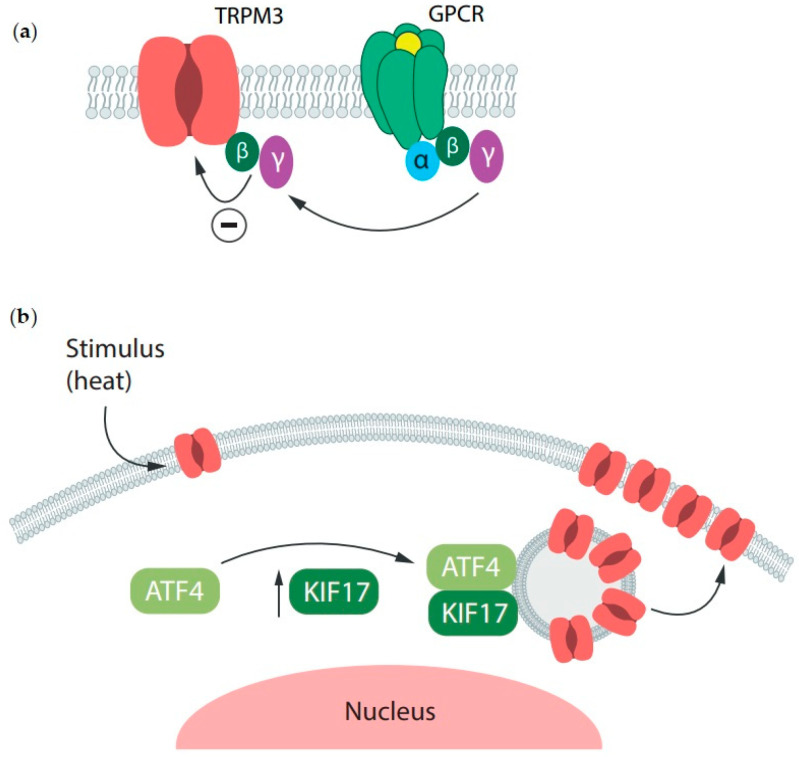
TRPM3 channel activity and expression are regulated by diverse proteins. (**a**) TRPM3 channels are molecular targets of the Gβγ subunit release after GPCR activation. (**b**) The activating transcription factor 4 (ATF4) increases TRPM3 channel membrane expression in a KIF17-dependent manner.

Activation of G-protein-coupled receptors, including µ-opioid, somatostatin, NPY, and GABAB receptors, inhibits TRPM3 channel activity through direct binding of the Gβγ subunit [23,69,87,88] (Figure 4a). Gβγ binds to a domain in the N-terminus of the TRPM3 protein, which is subject to alternative splicing [89]. In vivo, peripheral activation of these receptors with morphine, DAMGO, PYY, or baclofen attenuated nocifensive behavior induced by PS or CIM0216 injection [34,69,88]. Notably, activation of the µ-opioid receptor did not inhibit TRPV1 strongly, since DAMGO was ineffective in reducing the capsaicin-induced nocifensive behavior [87]. Additionally, systemic application of naloxone and BIIE0246, an inverse agonist for μ-opioid and NPY Y2 receptors, increased TRPM3 activity and TRPM3-induced nocifensive behavior, indicating a possible basal inhibitory effect on TRPM3 activity by these receptors [88].

G protein-coupled receptors (GPCRs) for inflammatory mediators like bradykinin (BK) and prostaglandins inhibit TRPM3 activity and reduce pain-related behaviors triggered by TRPM3 activation, as well as the heat hyperalgesia associated with inflammation [88]. However, as discussed in this section, inflammation increases sensitivity to heat, and this intracellular regulation of TRPM3 results in channel inhibition. To address this dilemma, Behrendt and colleagues discovered that calcium responses to PS were enhanced by the preceding application of BK in DRG neuron cultures expressing BK receptors. They found that BK receptor-mediated activation of intracellular signaling pathways involved diacylglycerol kinase, release of calcium from intracellular stores, and exocytosis. This indicates that signaling and trafficking pathways are involved in TRPM3 sensitization during inflammation. A possible explanation for this mechanism is that when bradykinin is applied before PS, little if any Gβγ is liberated during the BK stimulation, making it unavailable for TRPM3 inhibition.

Xie et al. [90] showed that a loss of expression of the activating transcription factor 4 (ATF4) results in a decrease in the membrane expression of TRPM3 without changes in total expression in DRG neurons. This indicates that ATF4 regulates TRPM3 not via gene expression, but by directly influencing TRPM3 trafficking. Moreover, heat stimulation promoted the ATF4/TRPM3 interaction and stabilized TRPM3 in the membrane of DRG neurons, contributing to thermal hypersensitivity, as evidenced by a reduction in thermal withdrawal thresholds in wild-type mice, but not in ATF4 knockdown or ATF4+/− mice. The authors also found that the motor protein kinesin KIF17 is involved in this ATF4-dependent TRPM3 trafficking (Figure 4b).

Finally, it is important to note that TRPM3 is involved in peptide release. Activation of TRPM3 in sensory nerve endings results in the release of CGRP in neurons innervating the skin and perivascular nerve fiber endings [91], as well as in neurons innervating the human ureter [92]. Thereby, TRPM3 channels can contribute to generating inflammation as an indirect means of affecting pain transmission.

### 3.3. TRPM8

TRPM8 channels are cationic channels permeable to divalent and monovalent cations. These non-selective Ca^2+^ channels are activated by cool temperatures (<23–28 °C), compounds that evoke a sensation of coolness, such as menthol, WS-12, and icilin, and voltage [93,94]. Interestingly, mice lacking TRPM8 channels have deficiencies in sensing moderately cold [40,95,96]. TRPM8 channels are highly expressed in sensory neurons in the trigeminal and dorsal root ganglia [97]. They can also be found in the retina, prostate tissue, bronchial and intestinal epithelium, bladder, and cornea, as well as some brain regions [16].

Colburn et al. [96] studied the role of TRPM8 in cold-evoked nocifensive responses. Using the CCI model, cold sensitivity was assessed by measuring responses to the application of acetone. They found that WT animals lift, shake, or lick the paw very briefly (<5 s) before ligation, and in TRPM8 KO animals this response was eliminated. Following CCI, WT mice showed marked sensitivity to acetone, with prolonged licking and shaking of the paw, indicating cold allodynia. In contrast, TRPM8 KO mice showed no significant increase in acetone sensitivity post-ligation. A similar result was seen in the CFA model, where TRPM8 animals did not show a response to the acetone test. These results suggest that TRPM8 may mediate most of the cold allodynia and hyperalgesia in WT animals.

The chemical activation of TRPM8 channels has been extensively studied. Intraperitoneal injection of icilin induces robust shivering and shaking behaviors in rodents, commonly referred to as “wet dog shakes” [98]. Notably, this effect is absent in TRPM8 KO animals [40,96]. Nevertheless, this response cannot be classified as nocifensive, as it is believed to be related to homeostatic thermal regulation and likely involves controlling core body temperature [99]. However, when icilin is injected locally into the hind paw, WT animals exhibit a significantly higher number of paw flinches compared to TRPM8 KO animals. Importantly, since icilin is also an agonist of TRPA1 channels [100], the authors measured its effect in TRPA1 KO animals and showed that these animals showed a similar number of flinches as the WT mice. On the other hand, in double knockout (DKO) animals for TRPM8 and TRPA1, the icilin-induced effect was barely noticeable and did not differ from that in TRPM8 KO animals. These data prove that intraplantar injection of icilin can provoke nocifensive responses that mimic pain behaviors induced by noxious temperatures and that this depends on TRPM8 channels.

After nerve injury, it has been reported that TRPM8 immunoreactivity and electrical activity in both DRG neurons and superficial lamina I and II of the dorsal horn are both enhanced compared to sham animals [101,102]. Moreover, the percentage of neurons sensitive to cold and menthol is significantly increased [101]. In contrasting results, in mice with CCI, a decrease in TRPM8 mRNA levels was found, despite persistent cold and menthol hypersensitivity [103]. However, in calcium imaging experiments, the authors found no alterations in the number of cold or menthol-responsive DRG neurons [103]. In the spinal nerve ligation (SNL) model in rats, TRPM8 levels measured by in situ hybridization were decreased. In the same model, using in vitro skin-nerve preparation, the authors found that the percentage of mechanosensitive C fibers responding to strong cold (0 °C) or icilin remained unchanged, while the percentage of mechanosensitive Aδ fibers responding to these stimuli nearly doubled [104]. In culture, increases in the number of cold-sensitive neurons obtained from injured or sham animals have also been observed [104,105]. It is possible that cold hypersensitivity may be affected by molecular remodeling of TRPM8-expressing cold nociceptors that became more excitable after nerve injury.

Given these conflicting results, it is not an easy task to correlate TRPM8 expression with the behavioral data. It is important to keep in mind that mRNA levels do not always correlate with the amount of protein. Also, the functionality of the channel is not reflected when the total amount of a protein is quantified.

In the CIPNP model, contrasting results have also been reported. Gauchan and colleagues [106] reported an upregulation of TRPM8 mRNA in DRGs from mice treated with oxaliplatin, and when animals were treated systemically with capsazepine, cold responses were reduced. In contrast, Knowlton et al. [107], when administered PBMC, found no effect on cold hypersensitivity; however, oxaliplatin-induced cold allodynia was absent in TRPM8 KO mice. Kawashiri et al. [108] reported that oxaliplatin and its metabolite, oxalate, increase TRPM8 expression at the mRNA level in DRGs from treated animals and cultured DRGs. They proposed that oxaliplatin causes an influx of intracellular calcium through L-type calcium channels, triggering the activation of the nuclear factor of activated T cells, which then upregulates TRPM8 expression. Another report revealed increased TRPM8 expression in medium-sized neurons of the DRG four days post-treatment [109]. However, a different study found no change in TRPM8 expression 90 h after treatment [110]. Oxaliplatin acutely (1 h post-treatment) increases TRPM8 currents, but this effect is lost after 24 h. This acute effect depends on the activity of the phospholipase C (PLC) pathway and the depletion of phosphatidylinositol 4,5-bisphosphate (PIP2). In the same line of evidence, it has been shown that paclitaxel, another chemotherapy drug, in vitro sensitizes the excitability of IB4(−) and IB4(+) sensory neurons due to augmenting the function of Nav1.8, TRPV1, and TRPM8 channels [111].

In conclusion, despite the high number of controversies, it seems that TRPM8 is at some level involved in cold allodynia since it acts as a key mediator of cold sensitivity and could be a promising target for managing neuropathic pain conditions characterized by cold-induced hypersensitivity.

TRPM8 channels appear to be regulated by Gαq linked GPCRs like Bradykinin and histamine receptors; however, this regulation is not free of controversies. Zhang et al. [112], proposed that Gαq pre-forms a complex with TRPM8 channels and directly inhibits their activity following the receptor activation. Moreover, Zhang [113] reported that Gαq binds to three arginine residues in the TRPM8 N terminus. Mutation of these three residues eliminates TRPM8 inhibition by Gαq and bradykinin while maintaining PIP2 sensitivity. The BK receptor also binds directly to TRPM8 and renders it insensitive to PIP2 depletion. On the other hand, Lui et al. [114] reported that Gαq inhibits the channel by decreasing its affinity for PIP2, thereby making the channel more sensitive to inhibition as PIP2 levels decrease. These differences were detailed and addressed by Liu and Rohacs [115], and they concluded that PIP2 is essential for TRPM8 function. In particular, the cryoEM structure of TRPM8-binding PIP2 offers a molecular-level understanding of how this lipid interacts with the channel [116]. When Gq-coupled receptors are activated, both the reduction in PIP2 levels and the direct binding of Gαq synergistically inhibit TRPM8, although the relative contribution of each pathway likely varies depending on the specific receptor involved. Finally, it is worth mentioning that there are multiple lines of evidence showing that PIP2 is necessary for menthol- and cold-induced TRPM8 activity, so finding the precise mechanism by which inflammatory mediators’ receptors regulate TRPM8 channels and if this regulation is receptor specific will help to understand TRPM8’s role in cold and pain sensation.

TRPM8 channels are also regulated by µ-opioid receptors. When morphine activates µ-opioid receptors, it induces rapid co-internalization of both the receptor and TRPM8 channels, resulting in cold analgesia in mice [117]. This effect can be reversed by naloxone. Additionally, TRPM8 KO animals show reduced morphine-induced cold analgesia. Interestingly, the authors did not observe changes in channel gating upon µ-opioid receptor activation, suggesting that the primary regulatory mechanism involves channel internalization. On the other hand, Gong and Jasmin [118] found, using patch-clamp recordings and Western blotting, that chronic morphine administration upregulates TRPM8 channels, whereas the selective TRPM8 antagonist RQ-00203078 blocked cold hyperalgesia in morphine-treated rats. Lastly, chronic morphine has been found to regulate TRPM8 channels through µ-opioid receptor signaling [119]. The authors reported that morphine induces menthol- and cold-sensitization of TRPM8 channels, an effect that can be blocked by inhibiting protein kinase C beta (PKCβ) or by mutating two PKC phosphorylation sites, S1040 and S1041. Additionally, TRPM8-expressing DRG neurons isolated from morphine-treated mice exhibited hyperexcitability.

Finally, TRPM8 channel activation may contribute to analgesia. Moderate cooling suppresses pain produced by other stimuli, while excessive cold induces pain. Similarly, lower doses of menthol and icilin can have analgesic effects. Proudfoot et al. [102] demonstrated that in the CCI model, applying TRPM8 activators peripherally or centrally, or even gently chilling the skin, has considerable analgesic effects. Peripherally applied icilin or menthol also activated slowly conducting afferents and inhibited the increased responses of individual dorsal horn neurons ipsilateral to nerve damage. Moreover, TRPM8 KO animals did not present a reversal of CCI-induced mechanical hypersensitivity by cooling [107]. Dhaka et al. [40] assessed the impact of temperature on biphasic pain behavior elicited by intraplantar formalin. Lowering the floor temperature from 24 °C to 17 °C in WT mice reduced both the first and second phases of the formalin-evoked response. In contrast, in TRPM8 KO animals, cold only inhibited the second phase. Thus, cold-induced analgesia involves both TRPM8-dependent and independent pathways. Lastly, Liu et al. [120] reported that menthol-evoked analgesia also involves endogenous opioid pathways since naloxone administered systemically reversed TRPM8-dependent analgesia.

These findings suggest that TRPM8 is the primary mediator of menthol-induced analgesia in both acute and inflammatory pain. TRPM8 is a crucial element of the endogenous cooling-induced analgesic mechanism, representing a promising therapeutic target for pain management.

## 4. TRPM Channels in Cancer Pain

TRPM channel family members are expressed in several types of cancer, and their role in oncogenic functions and signaling pathways has been extensively reviewed [121,122]. Different lines of evidence indicate that TRPM channels are promising targets for cancer therapy since their aberrant overexpression is required for cancer cell growth, survival, invasion, and epithelial–mesenchymal transition [121,122]. For example, TRPM7 expression in breast cancer has been correlated with significantly inferior recurrence-free survival and distant metastasis-free survival [123]. Its expression is required for the proliferation and cytotoxicity of human breast cancer cells due to a mechanism that includes Mg^+^ homeostasis and cell cycle regulation [26].

Every year, 10 million new patients are diagnosed with some form of cancer, and pain is one of the most common symptoms reported [124]. The development of chronic pain associated with cancer includes changes at cellular, tissue, and systemic levels. Tumor-secreted substances evoke phenotypic changes that sensitize nociceptors [125]. Moreover, cancer proliferation and metastasis can damage surrounding tissues and sensory fibers, causing changes that lead to neuropathic pain [126,127]. The complex interactions between tumors, primary afferent nociceptors, and the immune system contribute to the unpredictability of cancer, making pain management a significant challenge. Until now, cancer pain has been one of the most serious consequences of both cancer and the treatments used for treating it.

Despite the large body of evidence for the role of TRPM channels in cancer biology, there is little if any evidence of their role specifically in cancer pain. In addition to what we described above for chemotherapy agents such as oxaliplatin (which triggers pain states in part via TRP channels), it is important to mention that the pharmacological treatments often used in cancer patients directly or indirectly target TRPM channels. The World Health Organization (WHO) promotes an analgesic ladder for managing cancer pain. In this guideline, the recommendation is to start with nonsteroidal anti-inflammatory drugs (NSAIDs) and acetaminophen for mild pain. For mild-to-moderate pain, opioids like codeine or tramadol are suggested. Finally, in cases of moderate-to-severe pain, strong opioids such as morphine, fentanyl, and oxycodone are used.

NSAIDs are broad-range TRP channel blockers since they can block TRP channels from different subfamilies like TRPM2, TRPM3, TRPV4, and TRPC6. Klose et al. [128] reported that mefenamic acid blocks TRPM3 channels in the micromolar range (IC50 8.6 µM), whereas such a blocking effect is not observed in other TRP channel family members. Mefenamic acid is prescribed daily at doses of 500 to 1000 mg, leading to an average serum concentration of around 82.9 µM [129], which makes TRPM3 channels a non-conventional target of mefenamic acid for treating pain. Diclofenac is another NSAID that acts as an antagonist of human TRPM3 channels (pore blocker) and has been reported to act at micromolar levels [130].

As noted in the TRPM3 and TRPM8 sections, these channels are regulated by G-proteins. Therefore, part of the pain relief seen in cancer patients treated with opioids may result from this regulation following the activation of µ-opioid receptors.

## 5. Concluding Remarks

In this review, we discussed how TRPM2, TRPM3, and TRPM8 are linked to pain conditions. These channels are temperature sensitive but also respond to a wide range of other stimuli. This characteristic makes them difficult to study and likely contributes to the numerous controversies surrounding them. The field of study of ThermoTRP channels in pain is broad, and many details remain unclarified, however, despite these challenges, they hold significant promise as therapeutic targets for developing treatments for pain-related conditions.

TRPM2, TRPM3, and TRPM8 are promising targets for analgesic drug discovery. Significant progress has been made in the development of specific antagonists for TRPM3 and TRPM8, with some in clinical trials. BHC-2100, an orally administered, selective, potent, and peripherally restricted TRPM3 antagonist by Biohaven Pharmaceuticals, is currently in phase 1 trials for chronic pain [131].

PF-05105679, a TRPM8 antagonist from Pfizer, is also in phase 1. While it does not cause perceptible hypothermia, a single 900 mg dose showed efficacy in a cold pressor test comparable to oxycodone. Still, it led to unexpected adverse events, including an intolerable hot sensation in two subjects [132]. Another TRPM8 blocker, AMG-333 from Agmen Inc., entered phase 1 but failed due to adverse effects associated with TRPM8 antagonism [133].

Despite extensive drug discovery efforts, the promise of TRPM channel antagonists for pain relief remains elusive. So far, the only TRPM channel agonist approved by the FDA is menthol patches targeting TRPM8.

## Figures and Tables

**Table 1:** Classification of DRG neurons.

Fiber	Subtype	Positive for
A-fiber	Proprioceptors	Parvalbumin (a Ca^2+^ binding protein) Ntrk3high+Ntrk2 (neurotrophic tyrosine kinase)
	Aβ-rapid-adapting (RA)—low-threshold mechanoreceptors (LTMRs)	Ntrk3high+S100a16 (S100 calcium-binding protein A16)
	Aβ-field/slow-adapting (SA)—LTMRs	Ntrk3low+Ntrk2
	Aδ-LTMRs	Calca+Bmpr1b (calcitonin-related polypeptide Alpha+ bone morphogenetic protein receptor Type 1B)
	Aδ—high-threshold mechanoreceptors (HTMRs)	Calca+Smr2 (submaxillary gland androgen-regulated protein 2)
C-fiber	Nociceptors	Calca+Sstr2 (somatostatin receptor 2) Calca+Adra2a (adrenoceptor alpha 2A)
	Cold thermoreceptors	Trpm8 (transient receptor potential M8)
	cLTMRs	Th (tyrosine hydroxylase) Mrgprd (MAS-related GPR family member D
	Pruriceptors	Mrgpra3+Mrgprb4 (MAS-related GPR member A3 + Mas-related G protein-coupled receptor B4) Mrgpra3+Trpv1 (transient receptor potential V1) Sst (Somatostatin)
	Atf3 (activating transcription factor 3) cluster expressing injury-induced transcription factors	Atf3, Sox11 (SRY-box transcription factor 11), and Jun

## Data Availability

No new data were created or analyzed in this study.
